# Effect of prophylactic doses of enoxaparin on antifactor Xa activity confirmed by rotational thromboelastometry in critically ill patients: a preliminary prospective cohort study

**DOI:** 10.3389/fphar.2024.1498188

**Published:** 2025-01-15

**Authors:** Piotr F. Czempik, Artur Beberok

**Affiliations:** ^1^ Department of Anesthesiology and Intensive Care, Faculty of Medical Sciences in Katowice, Medical University of Silesia, Katowice, Poland; ^2^ Transfusion Committee, University Clinical Center of Medical University of Silesia in Katowice, Katowice, Poland; ^3^ Department of Pharmaceutical Chemistry, Faculty of Pharmaceutical Sciences in Sosnowiec, Medical University of Silesia, Katowice, Poland

**Keywords:** antifactor Xa activity, intensive care unit, low-molecular-weight heparin, pharmacodynamics, rotational thromboelastometry, enoxaparin

## Abstract

**Introduction:**

Critically ill patients present multiple risk factors for venous thromboembolism (VTE). Underdosing of antithrombotic medications can result in VTE even as bleeding remains a significant concern for critically ill patients. On the other hand bleeding, remaining a significant concern for the critically ill, can be worsend by overdosing of antithrombotic medications. The present study aimed to assess the effects of prophylactic doses of enoxaparin on antifactor Xa activity (anti-Xa) and rotational thromboelastometry (ROTEM) parameters in critically ill patients.

**Materials and methods:**

In this prospective single-center cohort study, the effects of enoxaparin were assessed via anti-Xa monitoring. Standard laboratory coagulation and ROTEM parameters were also determined using the same blood samples.

**Results:**

A total of 61 patients (42.6% women) were enrolled in this study, whose median age was 59.0 (interquartile range: 43.0–70.0) years. Based on anti-Xa, the effects of enoxaparin were normal in 35 subjects (57.4%); in 17 patients (27.9%), the anti-Xa troughs and/or peaks were higher than the prophylactic range; in 9 patients (14.7%), the anti-Xa peak was lower than the prophylactic range. There were differences among the anti-Xa groups with respect to some ROTEM parameters. No VTE was detected among the study subjects. In 3 subjects (4.9%), there were signs of bleeding, and these patients presented with longer thrombin times.

**Conclusion:**

Anti-Xa values may be within the prophylactic range in slightly more than half of the critically ill patients receiving enoxaparin at prophylactic doses. The dosing of low-molecular-weight heparin (LMWH) in critically ill patients may require individualization based on anti-Xa. Further studies are therefore required to establish a universal anti-Xa prophylactic range for LMWH, the timing of anti-Xa determination, and management of LMWH dosing.

## 1 Introduction

Critically ill patients hospitalized in the intensive care unit (ICU) often present multiple risk factors for venous thromboembolism (VTE) that are both patient-specific (e.g. cancer, history of VTE) and management-specific (e.g. sedation, mechanical ventilation, immobilization, presence of indwelling vascular catheters). VTE may also occur because of underdosing of antithrombotic medications; therefore, the majority of critically ill patients require pharmacological prophylaxis against VTE. However, bleeding has historically been a significant problem in critically ill patients hospitalized in the ICU, with almost 30% of the patients bleeding upon admission and approximately 10% of the patients experiencing at least one bleeding episode thereafter (Brown et al., 1988). Bleeding in ICU patients may occur because of various factors that are both patient-specific and iatrogenic, such as the effect of antithrombotic medications. Therefore, dosing of antithrombotic medications should be individualized and adjusted to a patient’s unique pathophysiology.

The most frequently used medications for VTE prophylaxis in patients hospitalized in the ICU are LMWHs. Direct oral anticoagulants (DOACs) or antiplatelet agents are not recommended for VTE prophylaxis in this patient population. A large meta-analysis assessing the effectiveness of VTE prophylaxis in critically ill patients confirmed that LMWHs should be the preferred choice (Fernando et al., 2022). It is of utmost importance to use appropriate doses of LMWHs to minimize the risks of both VTE and bleeding. Although manufacturers of LMWHs do not recommend prophylactic dose adjustment according to the patient’s weight or other factors that could influence the drug pharmacokinetics and potentially its pharmacodynamics, higher antifactor Xa activity (anti-Xa) has been considered in morbidly obese patients with body mass patients whose body mass index (BMI) ≥35 kg m^-2^ (Ludwig et al., 2011). Enoxaparin is a drug known to undergo renal elimination; hence, patients with acute kidney injury may experience accumulation of the drug and may require adjustment. An earlier case report from our institution showed that the pharmacodynamics of LMWHs in critically ill patients may be unpredictable (Czempik, 2024). Therefore, the current fixed-dose approach to VTE prophylaxis with LMWHs may not be optimal (Schizodimos et al., 2021).

Another unresolved issue involves the preferred method of monitoring VTE prophylaxis with LMWHs. The gold standard for monitoring the effect of LMWHs is anti-Xa. However, the decision of whether anti-Xa peak, trough, or both should be used for evaluations in critically ill patients remains unclear. Anti-Xa monitoring is not a widely available method, so other methods like rotational thromboelastometry (ROTEM) are attempted to be used for monitoring the effect of LMWH.

Taking the factors above into account, we expect that the pharmacodynamics of LMWHs may differ among critically ill patients. There are no extensive studies analyzing the pharmacodynamics of prophylactic doses of LMWHs among critically ill patients. A recent study in critically ill patients used the peak anti-Xa value for monitoring the effect of a standard dose of enoxaparin, which showed that anti-Xa was subprophylactic in approximately 40% of the patients (Baloo et al., 2021). Hence, the present study aimed to assess the effect of prophylactic doses of enoxaparin in critically ill patients hospitalized in the ICU using trough and peak anti-Xa values as well as a point-of-care (POC) test like ROTEM.

## 2 Materials and methods

This prospective single-center cohort study was conducted in a mixed medical-surgical ICU in a large academic medical center between November 2023 and July 2024. The principles of patient blood management (PBM) were implemented in the local ICU (Baloo et al., 2021). To minimize iatrogenic blood loss, low-volume test tubes and ROTEM were used.

### 2.1 Study population

The study population involved consecutive patients admitted to the ICU who required VTE prophylaxis. The risk of VTE was assessed using the Padua Prediction Score for Risk of VTE (Barbar et al., 2010) and Caprini Score for VTE (Caprini et al., 1991). The requirement for VTE prophylaxis corresponded to minimum scores of four and three points according to the former and latter scales, respectively. The exclusion criteria were contraindications for pharmacological VTE prophylaxis, namely severe coagulopathy, recent bleeding, and status directly post neurosurgical procedure.

### 2.2 Demographic and clinical data

The primary demographic and clinical data recorded were the sex, age, height, weight, BMI, body surface area (BSA), presence of comorbidities (chronic kidney disease, acute kidney injury, chronic liver disease, and acute liver disease), and therapeutic measures (continuous renal replacement therapy) having potential impact on bleeding tendency, type of LMWH, dose of LMWH, as well as antiplatelet agent name and dose. As steady state for prophylactic LMWH is achieved after five doses, the number of LMWH doses was recorded (Bates et al., 2012). As peripheral perfusion can impact subcutaneous drug adsorption, the norepinephrine (NE) dose used was recorded in terms of mcg kg^-1^ min^-1^. Episodes of VTE were also recorded. To diagnose VTE, clinical suspicion had to be confirmed with diagnostic imaging (Doppler ultrasound, chest computed tomography angiogram). The severity of disease was classified using the following three most common classification systems: simplified acute physiology score (SAPS) III (Moreno et al., 2005), acute physiology and chronic health evaluation (APACHE) II (Knaus et al., 1985), and sequential organ failure assessment (SOFA) (Vincent et al., 1996). The parameters ordered at the time of admission to the ICU and periodically during the ICU stay (standard laboratory panel) were recorded; these include complete blood count (hemoglobin, platelet count), standard laboratory tests (SLTs) of coagulation (prothrombin time, international normalized ratio, prothrombin activity, thrombin time, D-dimers, fibrinogen), creatinine, estimated glomerular filtration rate (eGFR), urea, blood urea nitrogen, total bilirubin, and arterial blood gas values. Creatinine clearance (CrCl) was calculated using the Cockroft–Gault formula (Cockcroft and Gault, 1976). It has been suggested that renal impairment and augmented renal function may impact the pharmacokinetics of LMWHs (Baptista et al., 2023).

### 2.3 VTE prophylaxis with LMWHs

For standard pharmacological VTE prophylaxis, the following were used in the local ICU: 40 mg of enoxaparin (Clexane, Sanofi-Aventis, Poland) once daily (OD) in patients with CrCl >30 mL min^-1^; 5000 international units (IU) of dalteparin (Fragmin, Pfizer Europe, Poland) OD in patients with CrCl ≤30 mL min^-1^. Patients receiving dalteparin for VTE prophylaxis were not included in the analyses because the pharmacokinetics and pharmacodynamics of different LMWHs vary, such that enoxaparin and dalteparin cannot be used interchangeably (Fareed et al., 2003).

### 2.4 Effect of prophylactic doses of enoxaparin

Studies have investigated the application of ROTEM for monitoring VTE prophylaxis with LMWHs in medical contexts (Fareed et al., 2003). In our study, the effect of enoxaparin were monitored using ROTEM (Werfen, Germany) through a panel assessing the intrinsic coagulation pathway (INTEM) and effect of heparin (HEPTEM), in addition to anti-Xa (Werfen, Germany). Anti-Xa is not affected by acute phase reactants (e.g. fibrinogen, factor VIII) or deficiencies in the coagulation factors. However, the limitations of the chromogenic anti-Xa assay include hemolysis, hyperbilirubinemia (total bilirubin >6.6 mg dL^-1^), hypertriglyceridemia (>360 mg dL^-1^), and reduced antithrombin activity (AT) (Hutt Centeno et al., 2019). Anti-Xa and ROTEM were performed using a blood sample from a single standard sodium-citrate-buffered 2.0-mL test tube (Vacutainer^®^, Beckton-Dickinson, Plymouth, United Kingdom). The blood left in the test tube after performing the POC INTEM and HEPTEM was used to determine the anti-Xa and SLTs of coagulation. These tests were performed on a day when a standard laboratory panel (basic biochemistry panel and basic coagulation panel) was ordered for a particular patient, so there was no additional blood loss associated with monitoring the effect of enoxaparin. The additional tests ordered were triglyceride concentration (standard laboratory panel biochemistry tube) and AT (standard laboratory panel coagulation tube). To assess the effect of enoxaparin, anti-Xa, ROTEM, and SLTs of coagulation were performed directly before the next scheduled enoxaparin dose (trough) as well as 4 h after subcutaneous injection of the next enoxaparin dose (peak). The 4-h time point for peak activity was chosen because the effect of the LMWH is highest at a point 3–4 h after subcutaneous injection (Levine et al., 1989). There are several reference ranges reported for anti-Xa in literature. The reference range of 0.2–0.5 IU mL^-1^ for anti-Xa at a point 4 h after administration of a prophylactic dose of enoxaparin (peak activity) was reported by the study by Weitz (2009). The effect of enoxaparin was classified as “normal” if the anti-Xa trough was <0.2 IU mL^-1^ and peak was in the range of 0.2–0.5 IU mL^-1^; if the anti-Xa trough was >0.2 IU mL^-1^ and/or the peak was >0.5 IU mL^-1^, then the effect was classified as “high”; if the anti-Xa peak was <0.2 IU mL^-1^, then the enoxaparin effect was classified as “low.” The diagnostic criteria for the effect of enoxaparin are presented in Table 1. As hypertriglyceridemia may affect the accuracy of the anti-Xa test, the triglyceride concentration was also recorded.

**TABLE 1:** Diagnostic criteria for the pharmacodynamic profiles of prophylactic doses of low-molecular-weight heparins used in this study.

Enoxaparin effect	Antifactor Xa activity trough	Antifactor Xa activity peak
Normal	<0.2 IU mL^–1^	0.2–0.5 IU mL^–1^
High	≥0.2 IU mL^–1^	>0.5 IU mL^–1^
Low	-	<0.2 IU mL^–1^

### 2.5 Monitoring of VTE and bleeding episodes

Clinical monitoring of the effects of enoxaparin was performed by recording episodes of confirmed VTE and signs of bleeding. Monitoring was carried out using the descriptive (location and recurrence of bleeding based on the hemorrhage measurement bleeding assessment tool) (Arnold et al., 2007) and severity of bleeding (WHO bleeding scale) tools (Miller et al., 1981).

### 2.6 Statistical analysis

The outcome viariable was the effect of prophylactic doses of enoxaparin based on the anti-Xa value. All statistical analyses were performed using licensed statistical software (18.0 Basic Edition, Stata, StataCorp LLC, College Station, United States). The Shapiro–Wilk test was used to determine the type of data distribution. The continuous variables were presented as medians (Me) and interquartile ranges (IQRs), whereas the categorical variables were presented as frequencies and percentages. The patients were categorized into groups in the context of pharmacodynamic profiles. Intergroup comparisons of the continuous variables were performed using analysis of variance (ANOVA) or Kruskal–Wallis test depending on the type of distribution. With the exception of anti-Xa, to determine the variables that could predict the effects of enoxaparin, multinomial logistic regression analysis was performed using the odds ratio (OR) and 95% confidence interval (CI) measures. To determine the effects of potential confounders, the likelihood ratio test was conducted for the model including potential confounders. Spearman’s rank correlation coefficient (ρ) was used to assess the associations between the anti-Xa, SLTs of coagulation, and ROTEM parameters.

### 2.7 Ethical approval

The study was designed so as to not increase the amount of blood lost for laboratory diagnostics. ROTEM analysis was routinely used in the local ICU to assess hemostasis and the effects of anticoagulants, and additional monitoring of the anti-Xa was performed using leftover blood (i.e. no extra blood loss). Therefore, the Bioethics Committee of the Medical University of Silesia in Katowice, Poland, decided that an ethics review was unnecessary for the present study (BNW/NWN/0052/KB/162/23).

## 3 Results

Eleven patients receiving dalteparin for VTE prophylaxis were excluded from the study. Thus, there were 61 patients in whom the effects of enoxaparin were analyzed; of these, 26 (42.6%) were women and 35 (57.4%) were men. Only 3 patients (4.9%) had chronic kidney disease, whereas 9 patients (14.7%) presented with acute kidney injury, and 2 patients (3.3%) had chronic liver disease. The clinical and laboratory characteristics of the study population are presented in Tables 2, 3. Eleven (15.3%) patients received low doses (≤150 mg/day) of acetylsalicylic acid. The median number of enoxaparin doses that the subjects received before analyses was over 3 (IQR: 3–4) days. Based on the anti-Xa value, the LMWH pharmacodynamic profile was “normal” in 35 subjects (57.4%); in 17 subjects (27.9%), the anti-Xa trough and/or peak were higher than the prophylactic range (“high”); in 9 subjects (14.7%), the anti-Xa peak was lower than the prophylactic range (“low”).

**TABLE 2:** Clinical and selected laboratory characteristics of the study population.

Parameter	Me (IQR)	Reference range
Age [years]	59.0 (43.0–70.0)	-
Body mass index [kg m^–2^]	26.2 (23.6–29.4)	18.5–24.9
SAPS III [points]	29.0 (20.0–37.0)	-
APACHE II [points]	8.0 (6.0–14.0)	-
SOFA [points]	3.0 (1.0–5.0)	-
Padua prediction score for risk of VTE [points]	7.0 (5.0–9.0)	
Caprini score for VTE [points]	10.0 (7.0–12.0)	
Creatinine [mg dL^–1^]	0.71 (0.56–0.96)	0.51–0.95 (women); 0.67–1.17 (men)
eGFR (MDRD) [mL min^–1^]	60.0 (60.0–60.0)	>60
Creatinine clearance (Cockroft–Gault) [mL min^–1^]	112.0 (75.0–152.0)	88–128 (women); 97–137 (men)
Blood urea nitrogen [mg dL^–1^]	29.1 (19.5–42.5)	7.9–20.0
Urea [mg dL^–1^]	62.2 (41.8–91.0)	16.6–48.5
Bilirubin [mg dL^–1^]	0.3 (0.2–0.5)	0.3–1.2
Triglycerides [mg dL^–1^]	178.0 (115.0–276.0)	<200
Hemoglobin [g L^–1^]	103.0 (81.0–117.0)	115–150 (women); 135–165 (men)

APACHE II, acute physiology and chronic health evaluation II; eGFR (MDRD), glomerular filtration rate estimated with modification of diet in renal disease formula; IQR, interquartile range; Me, median value; SOFA, sequential organ failure assessment; SAPS III, simplified acute physiology score III; VTE, venous thromboembolism.

**TABLE 3:** Hemostatic parameters in the study population.

Parameter	Me (IQR)	Reference range
Platelets [× 10^3^ μL^−1^]	268.0 (189.0–357.0)	130–400
Antithrombin [%]	116.0 (88.0–135.0)	75.0–120.0
Prothrombin time trough [s]	12.4 (11.6–13.3)	9.4–12.5
Prothrombin time peak [s]	12.6 (11.9–13.6)	-
International normalized ratio trough	1.03 (0.96–1.10)	0.80–1.20
International normalized ratio peak	1.04 (0.98–1.13)	-
Prothrombin activity trough [%]	89.0 (80.0–99.0)	80.0–120.0
Prothrombin activity peak [%]	87.0 (77.0–95.0)	-
aPTT trough [s]	30.0 (26.5–33.1)	25.4–36.9
aPTT peak [s]	34.7 (30.2–38.0)	-
Thrombin time trough [s]	15.0 (14.1–16.5)	10.3–16.6
Thrombin time peak [s]	15.9 (15.1–17.8)	-
D-dimers trough [ng mL^−1^]	2450.0 (1248.0–5482.0)	<500
D-dimers peak [ng mL^−1^]	2560.0 (1162.0–5272.0)	-
Fibrinogen trough (Clauss) [mg dL^−1^]	600.0 (460.0–757.0)	200–393
Fibrinogen peak (Clauss) [mg dL^−1^]	598.0 (423.0–786.0)	-
Antifactor Xa activity trough [IU mL^−1^]	0.09 (0.05–0.18)	<0.2
Antifactor Xa activity peak [IU mL^−1^]	0.29 (0.23–0.41)	0.2–0.5
INTEM CT trough [s]	190.0 (177.0–213.0)	100–240
INTEM CT peak [s]	211.0 (193.0–236.0)	-
HEPTEM CT trough [s]	178.0 (161.0–195.0)	100–240
HEPTEM CT peak [s]	182.0 (166.0–194.0)	-
INTEM CT–HEPTEM CT trough [s]	14.0 (6.0–27.0)	-
INTEM CT–HEPTEM CT peak [s]	31.0 (24.0–45.0)	-

aPTT, activated partial thromboplastin time; CT, clotting time; IQR, interquartile range; Me, median value.

Intergroup comparisons for the clinical and laboratory characteristics among these groups are presented in Table 4. The intergroup comparison were performed using the Kruskal–Wallis test. The only parameters that significantly varied between these three groups were INTEM CT trough as well as difference between INTEM CT trough and HEPTEM CT trough. Using logistic regression, the INTEM CT trough was found to predict normal (OR: 0.98, 95% CI: 0.96–0.99, *p* = 0.03) and high (OR: 1.02 95% CI: 1.00–1.04, *p* = 0.02) enoxaparin effect; however, it could not predict the low enoxaparin effect (OR: 1.00, 95% CI: 0.98–1.02, *p* = 0.79). The difference between INTEM CT trough and HEPTEM CT trough could not predict normal (OR: 0.97, 95% CI: 0.94–1.00, *p* = 0.05), high (OR: 1.02, 95% CI: 0.99–1.06, *p* = 0.10), or low (OR: 1.01, 95% CI: 0.98–1.04, *p* = 0.58) enoxaparin effect.

**TABLE 4:** Comparisons between groups with different antifactor Xa activity levels.

Parameter	Antifactor Xa activity			*p*-value
	Normal, Me (IQR) n = 35 (57.4%)	High, Me (IQR) n = 17 (27.9%)	Low, Me (IQR) n = 9 (14.7%)	
Age [years]	58.0 (43.0–70.0)	56.0 (34.0–70.0)	66.0 (45.0–72.0)	0.54
Body mass index [kg m^−2^]	26.0 (23.8–27.4)	26.4 (23.1–29.4)	31.4 (23.4–33.1)	0.62
SAPS III [points]	25.0 (19.0–36.0)	30.0 (22.0–41.0)	29.0 (22.0–34.0)	0.56
APACHE II [points]	8.0 (5.0–13.0)	9.0 (2.0–17.0)	9.0 (7.0–10.0)	0.71
SOFA [points]	2.0 (1.0–5.0)	3.0 (1.0–6.0)	2.0 (1.0–4.0)	0.89
Creatinine [mg dL^−1^]	0.7 (0.6–0.9)	0.7 (0.5–1.2)	0.9 (0.7–1.4)	0.52
eGFR (MDRD) [mL min^−1^]	60.0 (60.0–60.0)	60.0 (60.0–60.0)	60.0 (48.5–60.0)	0.23
CrCl (Cockroft–Gault) [mL min^−1^]	112.0 (81.0–152.0)	109.0 (73.0–167.0)	121.0 (60.0–143.0)	0.89
Blood urea nitrogen [mg dL^−1^]	29.1 (19.5–38.0)	26.7 (17.9–45.3)	31.0 (22.4–47.7)	0.75
Urea [mg dL^−1^]	62.2 (41.8–81.4)	57.2 (38.3–96.9)	66.4 (47.9–102.0)	0.75
Bilirubin [mg dL^−1^]	0.3 (0.2–0.5)	0.3 (0.2–0.4)	0.3 (0.2–0.6)	0.86
Triglycerides [mg dL^−1^]	185.0 (116.0–284.0)	154.0 (112.6–258.0)	166.0 (115.0–228.0)	0.64
Hemoglobin [g L^−1^]	99.0 (79.0–120.0)	106.0 (94.0–119.0)	89.0 (72.0–106.0)	0.29
Platelets [× 10^3^ μL^−1^]	277.0 (192.0–422.0)	231.0 (186.0–325.0)	307.0 (174.0–352.0)	0.72
Antithrombin [%]	118.0 (91.0–135.0)	125.0 (112.0–138.0)	86.0 (47.0–107.0)	0.06
Prothrombin time trough [s]	12.4 (11.7–13.3)	11.8 (11.5–12.8)	13.2 (11.6–14.4)	0.32
Prothrombin time peak [s]	13.1 (11.9–13.6)	12.4 (11.7–12.9)	12.9 (11.5–14.9)	0.56
International normalized ratio trough	1.03 (0.97–1.10)	0.97 (0.95–1.06)	1.07 (0.95–1.19)	0.30
International normalized ratio peak	1.08 (0.98–1.13)	1.03 (0.97–1.07)	1.07 (0.95–1.23)	0.56
Prothrombin activity trough [%]	89.0 (80.0–98.0)	96.0 (85.0–100.0)	81.0 (71.0–99.0)	0.32
Prothrombin activity peak [%]	82.0 (77.0–95.0)	89.0 (84.0–98.0)	84.0 (68.0–100.0)	0.56
aPTT trough [s]	28.8 (26.4–32.4)	31.8 (27.4–36.4)	30.5 (27.0–32.5)	0.51
aPTT peak [s]	35.0 (30.6–37.4)	34.3 (29.2–38.0)	35.1 (29.5–40.4)	0.94
Thrombin time trough [s]	14.7 (14.1–15.8)	16.1 (15.0–17.0)	14.7 (14.2–16.5)	0.11
Thrombin time peak [s]	15.8 (15.4–16.8)	16.5 (15.6–18.4)	14.9 (14.4–18.3)	0.28
D-dimers trough [ng mL^−1^]	2,794.0 (1248.0–5877.0)	1,606.0 (964.0–4868.0)	2,613.0 (1655.0–5482.0)	0.42
D-dimers peak [ng mL^−1^]	2,727.0 (1339.0–5272.0)	1,668.0 (1011.0–4849.0)	2,560.0 (2173.0–5308.0)	0.43
Fibrinogen trough (Clauss) [mg dL^−1^]	652.0 (460.0–757.0)	521.0 (460.0–705.0)	715.0 (399.0–850.0)	0.82
Fibrinogen peak (Clauss) [mg dL^−1^]	633.0 (430.0–791.0)	550.0 (403.0–638.0)	633.0 (392.0–791.0)	0.55
INTEM CT trough [s]	**183.0 (164.0–204.0)**	**213.0 (189.0–222.0)**	**199.0 (186.0–208.0)**	**<0.01**
INTEM CT peak [s]	204.0 (192.0–232.0)	232.0 (204.0–241.0)	213.0 (182.0–226.0)	0.12
HEPTEM CT trough [s]	173.0 (154.0–195.0)	182.0 (167.0–205.0)	181.0 (169.0–186.0)	0.26
HEPTEM CT peak [s]	176.0 (163.0–185.0)	185.0 (177.0–196.0)	182. (174.0–194.0)	0.08
INTEM CT–HEPTEM CT trough [s]	**8.0 (3.0–21.0)**	**23.0 (18.0–30.0)**	**18.0 (13.0–22.0)**	**0.02**
INTEM CT–HEPTEM CT peak [s]	31.0 (18.0–44.0)	36.0 (29.0–51.0)	31.0 (16.0–36.0)	0.21

aPTT, activated partial thromboplastin time; APACHE II, acute physiology and chronic health evaluation II; CrCl, creatinine clearance; CT, clotting time; eGFR (MDRD), glomerular filtration rate estimated with modification of diet in renal disease formula; IQR, interquartile range; Me, median value; SOFA, sequential organ failure assessment; SAPS III, simplified acute physiology score III. In bold: statistically significant differences according to the Kruskal–Wallis test.

**FIGURE 1:**
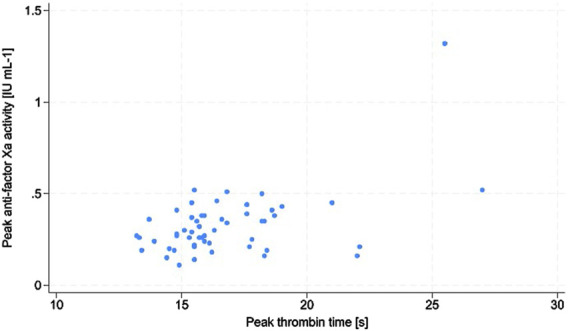
Scatter plot of the associations between peak antifactor Xa activity and peak thrombin time.

The two most important confounding factors, namely CrCl and BMI, were shown to not have any impacts on the enoxaparin effect in the study subjects. The *p*-values of the logistic regression test for the model including CrCl was 0.88 and that for the model including BMI was 0.35. There were no episodes of VTE noted during the study. In 3 patients (4.9%), signs of bleeding were present (WHO bleeding scale grade 2); these were observed as ecchymoses, bloody postoperative drainage, and gross hematuria. Intergroup comparisons between patients with and without bleeding showed significant differences in the thrombin time (TT) trough (Me: 18.2 s with IQR: 18.2–18.3 s vs. Me: 15.0 s with IQR: 14.1–16.5 s, *p* = 0.03). There were positive associations between TT trough and anti-Xa trough (ρ = 0.30, *p* = 0.02), AT and anti-Xa trough (ρ = 0.26, *p* = 0.04), as well as TT peak and anti-Xa peak (ρ = 0.34, *p* = 0.01) (Figure 1).

## 4 Discussion

In the present study, we aimed to assess the effect of prophylactic doses of enoxaparin in critically ill patients hospitalized in the ICU using several tests, namely anti-Xa (reference method), ROTEM (POC global hemostasis assay), and SLTs (conventional coagulation tests). Anti-Xa has been used in some studies to monitor the prophylactic effect of LMWHs in critically ill patients. Jochberger et al. (2005) performed a prospective cohort study in critically ill patients (n = 62); these patients received certoparin 3000 IU once or twice daily, and the prophylactic range used was 0.1–0.3 IU mL^-1^. Anti-Xa values were determined after the second LMWH dose, which could be too early for achieving stable anticoagulant effect, which usually require approximately 3–5 doses. Despite using a lower prophylactic reference range, only in 28% (OD dosing) and 47% (twice daily dosing) of patients showed anti-Xa peaks within this range, which is very similar to the proportion of patients who attained prophylactic anticoagulation in our study. The authors of the earlier study also showed that low AT was independently associated with low anti-Xa values (Jochberger et al., 2005). In our study, differences in AT between the enoxaparin effect groups were close to statistical significance.

Niziolek et al. (2024) performed another study among critically ill patients, where the patients received 30 mg of enoxaparin twice daily; the prophylactic reference range used in this work was the same as that in our present study (0.2–0.5 IU mL^-1^). Although the enoxaparin dose was higher than that in our study and reference range used was the same, only 43% of patients achieved target anticoagulation. This could be explained by the fact that the majority of these patients were trauma cases in whom there is an even greater prothrombotic tendency (Niziolek et al., 2024).

Surprisingly, the enoxaparin effect of more than a quarter of the patients in our study was high. The anti-Xa values should be lower than the prophylactic range considering that all the patients in our study received low prophylactic doses of enoxaparin. We noticed a similar issue in an oncologic patient hospitalized in our department following a major surgical procedure. Although we expected a low enoxaparin effect, the anti-Xa trough was much higher and could potentially lead to severe complications during epidural catheter removal (Czempik, 2024).

The prophylactic range for LMWHs is an unresolved issue. Depending on the range chosen, different conclusions may be drawn regarding the efficacy of the LMWHs. It is yet to be determined whether the trough or peak anti-Xa values should be used; the answer to this question might probably rest on an ongoing randomized controlled trial (Wang et al., 2023). In general, several factors can influence the efficacy of LMWH-based VTE prophylaxis. An earlier systematic review on anti-Xa monitoring in obese patients receiving LMWHs in prophylactic and therapeutic doses showed that anti-Xa monitoring is not required to ascertain clinical effectiveness (Egan and Ensom, 2015). However, obesity is a major risk factor for VTE owing to decreased levels of physical activity, elevated intraabdominal pressures, chronic inflammation, and decreased fibrinolysis (Hotoleanu, 2020). In the study by Freeman et al. (2012), the optimal prophylactic dose of enoxaparin in patients with BMIs ≥40 kg m^-2^ was reported to be 0.5 mg kg^-1^ of the actual bodyweight based on the anti-Xa values (0.2–0.5 IU mL^-1^). In our study, there were only two patients with BMIs ≥40 kg m^-2^, and one of these patients did not achieve prophylactic anti-Xa range (data not shown).

Although there were no VTE episodes in our study, three patients experienced non-severe bleeding (WHO grade 2 bleeding). There were no differences in the anti-Xa troughs or peaks between the bleeding and non-bleeding patients. The only parameters that differed between these groups was the TT. The common bleeding sites in critically ill patients are the upper gastrointestinal tract (35%), urinary tract (25%), and lung and skin (10%) (Brown et al., 1988). In another study, Arnold et al. (2007) reported the following bleeding sites: gastrointestinal bleeding (52%), insertion sites of peripheral and central vascular catheters (40%), endotracheal tubes (16%), and surgical wounds (14%). The typical bleeding sites or modes in our study were through urine, subcutaneous tissue, and surgical wounds.

One of the strengths of our study is that we use three different methods to assess the effects of enoxaparin. The timing of determination was extremely precise, with the trough determinations being made mere seconds before LMWH administration and peak determinations being made precisely 4 h thereafter. Another strength of this study is the fact that the episodes of VTE and bleeding were recorded. Aside from the primary aim of this study, we performed some interesting secondary analyses, such as finding the associations between SLTs of coagulation and anti-Xa.

Our study also has some potential limitations. First, this was a single-center study; therefore, the results may not be applicable to other settings with different patient populations. However, this is an obvious characteristic of a preliminary study. Second, the number of patients enrolled in the study was a limitation; however, the number of patients was considered adequate given this type of preliminary study. Third, the anti-Xa prophylactic range used in this study could be considered a limitation as there are other ranges reported in literature. However, we assumed a liberal approach and decided on the broadest range of values reported; this was partly dictated by the fact that even with a higher upper threshold for the reference range (i.e., 0.5 IU mL^-1^), pharmacological prophylaxis should be safe in terms of bleeding. Fourth, another limitation of this study is the lack of longitudinal data what may have affected the reported prevalences of both VTE and bleeding episodes. The last limitation is the fact that some patients (13.1%) were receiving low doses (≤150 mg/day) of acetylsalicylic acid that could have impacted the ROTEM results.

## 5 Conclusion

The observed anti-Xa values were within the prophylactic range in slightly over half of the patients receiving prophylactic doses of enoxaparin in this study; the remaining anti-Xa values were either higher or lower than the prophylactic range. Only the INTEM CT troughs significantly differed between the enoxaparin effect groups, which could be used to predict normal and high enoxaparin effects. Based on the anti-Xa and INTEM CT trough values, VTE prophylaxis with LMWHs in critically ill patients may require individualization. Larger studies with more diverse cohorts of patients are thus required to establish universal standards for the anti-Xa prophylactic range for LMWHs, timing of anti-Xa determination, and management of LMWH dosing.

## Data Availability

The raw data supporting the conclusions of this article will be made available by the authors, without undue reservation.
